# DNA methylation patterns of LINE-1 and *Alu* for pre-symptomatic dementia in type 2 diabetes

**DOI:** 10.1371/journal.pone.0234578

**Published:** 2020-06-11

**Authors:** Chanachai Sae-Lee, Julien De Biasi, Natassia Robinson, Timothy M. Barrow, John C. Mathers, Georgios Koutsidis, Hyang-Min Byun

**Affiliations:** 1 Population Health Sciences Institute, Newcastle University, Newcastle upon Tyne, United Kingdom; 2 Research division, Faculty of Medicine, Siriraj Hospital, Mahidol University, Bangkok, Thailand; 3 Department of Applied Sciences, Faculty of Health and Life Sciences, Northumbria University, Newcastle upon Tyne, United Kingdom; 4 Institute of Health & Society, Newcastle University, Newcastle upon Tyne, United Kingdom; 5 Faculty of Health Sciences and Wellbeing, University of Sunderland, Sunderland, United Kingdom; University of Helsinki, FINLAND

## Abstract

The identification of early markers of dementia is important for higher-risk populations such as those with type 2 diabetes (T2D). Retrotransposons, including long interspersed nuclear element 1 (LINE-1) and *Alu*, comprise ~40% of the human genome. Although dysregulation of these retrotransposons can induce aberrant gene regulation and genomic instability, their role in the development of pre-symptomatic dementia (PSD) among T2D patients is unknown. Here, we examined locus-specific changes in LINE-1 and *Alu* methylation in PSD and the potential to offset these changes via supplementation with folate and vitamin B_12_. We interrogated DNA methylation patterns corresponding to 22,352 probes for LINE-1 and *Alu* elements using publicly-available Illumina Infinium 450K methylation datasets from i) an 18-month prospective study in 28 T2D patients (GSE62003) and ii) an intervention study in which 44 individuals were supplemented with folic acid (400 μg/day) and vitamin B_12_ (500 μg/day) over two years (GSE74548). We identified 714 differentially methylated positions (DMP) mapping to retrotransposons in T2D patients who developed PSD in comparison to those who did not (P_FDR_ < 0.05), comprised of 2.4% (228 probes) of all LINE-1 probes and 3.8% (486 probes) of all *Alu* probes. These loci were enriched in genes with functions related to Alzheimer's disease and cognitive decline, including *GNB5*, *GNG7* and *PKN3* (p < 0.05). In older individuals supplemented with folate/vitamin B_12_, 85 (11.9%) PSD retrotransposon loci showed significant changes in methylation (p < 0.05): participants with the *MTHFR* CC genotype predominantly showed hypermethylation at these loci, while hypomethylation was observed more frequently in those with the TT genotype. In T2D patients, LINE-1 and *Alu* elements are differentially methylated in PSD in a locus-specific manner and may offer clinical utility in monitoring risk of dementia. Further work is required to examine the potential for dietary supplementation in lowering the risk of PSD.

## Introduction

Type 2 diabetes mellitus (T2D) is a metabolic disease affecting more than 200 million people worldwide in 2010, and global prevalence is predicted to increase to more than 400 million people by 2030 [1]. T2D is a recognised risk factor for dementia and for cognitive dysfunction in older people, affecting domains of processing speed, cognitive flexibility, learning and memory [2–7]. Alzheimer's disease (AD) is the most common cause of dementia and its development is associated with insulin resistance in the brain [8]. Up to 80% of AD patients have either impaired fasting blood glucose or T2D, and these conditions are associated with cell death in the brain and loss of β-cells in the pancreas [9].

To date, the characteristics of T2D patients who go on to develop dementia are unclear. Epigenetic changes occur during cognitive dysfunction and the development of dementia in T2D patients and, therefore, have potential as disease biomarkers. Retrotransposons, including long interspersed nuclear element 1 (LINE-1) and *Alu*, comprise approximately 40% of the human genome [10]. There are one-million copies of *Alu* and over 500,000 copies of LINE-1 in the human genome, which can replicate and integrate themselves into new locations throughout the genome [11–13]. The evolutionary age of these elements, that is the timing of their insertion into the human genome, can be estimated through sequence variants that facilitate their categorisation into subfamilies. Younger LINE-1 and *Alu* subfamilies have retained the ability to retrotranspose within the human genome [12, 14], and incorporation of these elements can interfere with the expression of adjacent genes, disrupting regulatory sequences of exon-intron interactions [15–17]. Aberrant activity of transposable elements has been implicated in neurological disorders, such as multiple sclerosis and AD, and can promote neuronal loss [18–20]. Furthermore, LINE-1 and *Alu* methylation has been widely measured as surrogate marker for global DNA methylation [15, 21–25]. Hypomethylation of these elements is associated with genomic instability [26] and occurs in multiple diseases including cancer [15, 22, 27] and AD [28]. Hypomethylation of retrotransposons is an essential factor promoting retrotransposition and becomes more common during ageing [15, 29, 30]. Blood methylation signatures of pre-symptomatic dementia (PSD) in older individuals with T2D have been identified, including differential methylation of loci mapping to genes linked to dementia [31]. Additionally, retrotransposons, including LINE-1 and *Alu*, are active in the human central nervous system throughout life [32, 33]. Therefore, we hypothesise that DNA methylation changes within LINE-1 or *Alu* elements are potential novel markers of early-stage dementia in T2D.

Folate and vitamin B_12_ are the source of coenzymes in one-carbon metabolism [34], and are involved in the maintenance of normal epigenetic patterns [35] by generating *S*-adenosylmethionine (SAM), the main methyl group donor for DNA methylation. Low serum concentrations of vitamin B_12_ and folate are observed frequently in AD patients [36] and can disrupt one-carbon metabolism and, therefore, SAM production [37]. Folate is essential for the development of the central nervous system, and folate insufficiency leads to high circulating concentrations of homocysteine [38], which are associated with cardiovascular disease [39, 40], venous thrombosis, cancer, psoriasis, T2D [39], renal and thyroid dysfunction [41], and with the development of dementia and AD [42, 43]. The well-characterised polymorphism within the methylenetetrahydrofolate reductase (MTHFR) gene at position 677 (C677T) results in a dose-dependent decrease in enzyme activity [44], leading to reduced SAM production, increased serum homocysteine and decreased serum folate concentration [45, 46]. However, the potential for intervention with nutrients involved in one-carbon metabolism to offset the epigenetic changes associated with PSD in T2D patients is unknown.

DNA methylation patterns are well known to display tissue-specific differences [47], but most loci show similar DNA methylation levels across a wide variety of tissue types. Equally, epigenetic changes in disease and in response to external stimuli may be unique to target tissues or may be more widespread throughout the body, and this is an important consideration when seeking to identify blood-based epigenetic biomarkers of disease. Changes in DNA methylation measured in the blood have been reported in response to a variety of environmental exposures [48, 49] and lifestyles [50], and also in the blood of patients with variety of pathologies [51, 52], but their role in disease aetiology can be complex. For example, differences in DNA methylation patterns observed in the blood of cancer patients may relate to changes in leukocyte composition of the blood as part of the body’s immune response to the tumour [53]. In the case of dementia, there is evidence that epigenetic marks in blood can serve as biomarkers of early disease, which may be related to brain-blood barrier leakage in the early stages of Alzheimer disease [54]. Most blood-based studies have identified differences in DNA methylation associated with pre-symptomatic dementia (PSD) but with little correlation to changes in brain tissue [55], which may imply that they are associated with risk factors for the disease (e.g. chronic inflammation) rather than informing upon epigenetic changes in the brain. Indeed, some of the genes whose differential methylation with PSD are unique to blood have roles in inflammatory processes [56]. However, some genes show disruption in both blood and brain tissue, such as differential methylation of the *OXT* gene that encodes the neuropeptide oxytocin [56]. Furthermore, in an analysis of blood samples taken from older individuals with T2D, genes with known functional relevance to dementia showed DNA methylation changes that were associated with PSD [31].

In this study, publicly-available Illumina Infinitum 450K methylation datasets were utilised to examine DNA methylation of retrotransposons in blood of older T2D patients with and without PSD, and to examine the potential of supplementation with folic acid and vitamin B_12_ to offset the epigenetic changes observed in PSD.

## Materials and methods

### Study design

**Fig 1:**
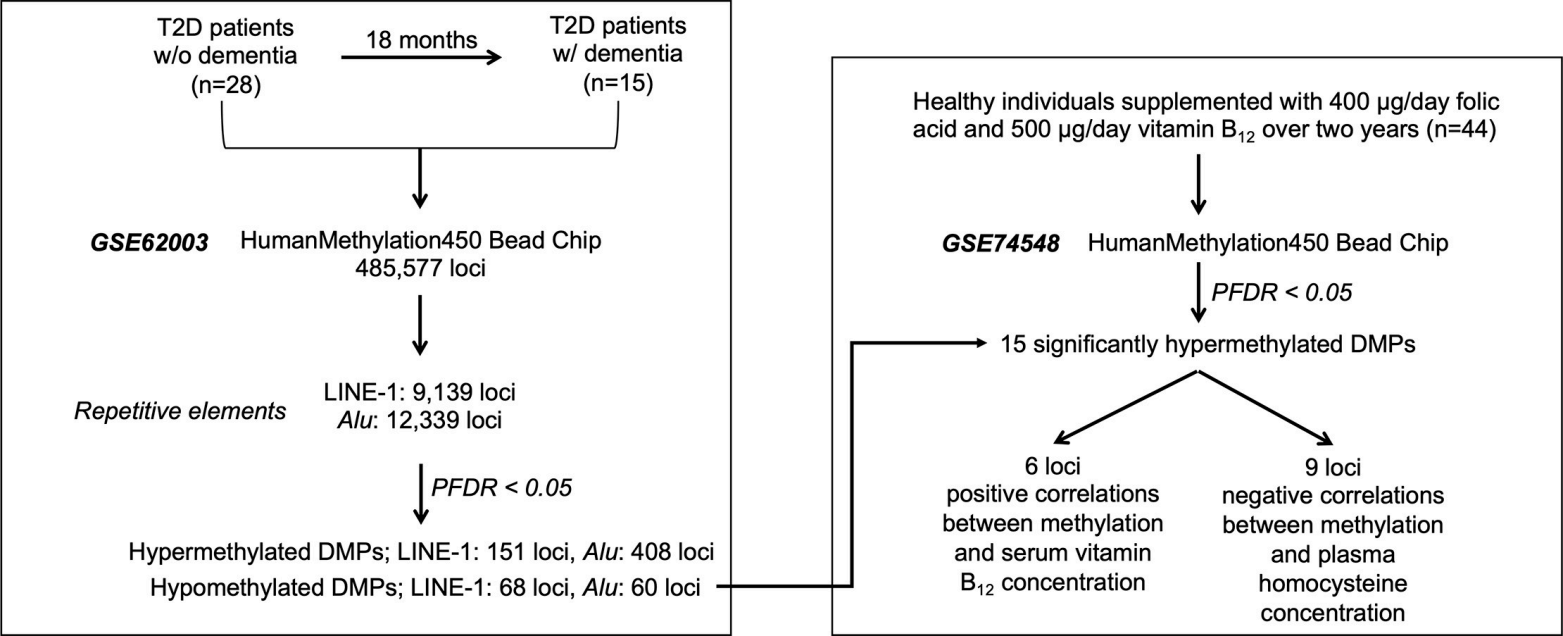
Study design.

Publicly available Illumina Infinium 450K datasets from two studies were used to analyse retrotransposon methylation (Fig 1). We used DNA methylation data from the Israel Diabetes and Cognitive Decline (IDCD) study (GSE62003)[31] to identify epigenetic changes in PSD. This study included 36 older T2D patients (mean age = 73.9 years) with a clinical dementia rating (CDR) of 0.0 at baseline, of whom 18 showed a decline in cognition to CDR = 0.5 at 18-months follow-up, while 18 controls remained without dementia (CDR = 0). T2D with PSD patients was age, gender, education, and HbA1C matched to the T2D without PSD (S1 Table). Secondly, data from an intervention study of folic acid and vitamin B_12_ supplementation (GSE74548)[57] were used to determine the effect of these nutrients on methylation of retrotransposons. This study was a part of the B-PROOF study and recruitment criteria have been described in previous study [58]. Briefly, 44 older participants (mean age = 70.8 years) were supplemented with 400 μg/day folic acid and 500 μg/day vitamin B_12_ over two years and 43 participants constituted the placebo group. Both groups were non-smokers (25 and 31 former smokers in supplemented and placebo groups, respectively). No excessive alcohol users were recruited. Levels of the C-reactive protein (CRP) at baseline was ≤ 10 mg/L. In both studies, DNA was extracted from peripheral blood samples and DNA methylation was analysed using the Illumina Infinium 450K methylation microarray platform at two time points (baseline and 18-month follow-up in the IDCD study; and baseline and two-year follow-up in the folic acid and vitamin B_12_ intervention study). *MTHFR* genotype data were available for the dietary intervention study (22 participants with 677CC and 22 participants with 677TT). Ethical approval for the longitudinal IDCD study was granted by the Institutional Review Board of Mount Sinai and the Helsinki committees of Sheba and MHS [59], and for the dietary intervention study by the Academic Hospital Maastricht [57].

### Data analysis

Datasets from the intervention study and IDCD study were normalised using the wateRmelon method, respectively [31, 57]. Data from the two studies were analysed separately and therefore normalisation of the two datasets together was not required. After data filtering and quality control, 15 subjects who developed dementia and 13 who had normal cognitive function were retained from within the IDCD dataset. Probes on the Illumina Infinium 450K methylation microarray mapping to LINE-1 and *Alu* elements were extracted using RepeatMasker and categorised according to evolutionary age of subfamilies into oldest (L1M; *Alu*J), intermediate (L1P and L1PB; *Alu*S), and youngest (L1HS and L1PA; *Alu*Y) [20]. Top differentially methylated positions (DMPs) were identified in PSD patients by paired-sample *t*-tests and ANOVA, with correction for multiple hypothesis testing using the Benjamini-Hochberg method and significance defined as P_FDR_ < 0.05. The genomic locations (TSS200, TSS1500, 5’UTR, 3’ UTR, 1^st^ Exon, and gene body) of probes were identified through the Illumina annotation. The pathway analysis with significantly differentially methylated loci was performed using WebGestalt (WEB-based Gene SeT AnaLysis Toolkit) [60].

### Statistical analysis

Differentially methylated loci were identified by paired-sample *t*-tests and ANOVA. Paired *t*-test was used to identify significant changes from baseline in DNA methylation of retrotransposons in both PSD and supplementation datasets. Fisher’s exact test was used to identify enrichment by genomic location and evolutionary age of retrotransposons. ANOVA was conducted to assess changes in DNA methylation by genomic location. Correlations between DNA methylation and plasma homocysteine and serum vitamin B_12_ concentrations were assessed by Pearson’s correlation coefficient. The impact of *MTHFR* genotype upon the effects of folate supplementation was assessed by paired t-test. All analyses were performed using IBM SPSS statistical software program (version 24) and R Studio (version 1.1.442). Data are presented as means ± SD unless otherwise stated. Probabilities (P) were adjusted for multiple hypothesis testing by the Benjamini-Hochberg method, with P_FDR_ < 0.05 considered significant.

## Results

### Distribution of retrotransposon (LINE-1 and *Alu*) probes by subfamily and genomic location

**Fig 2:**
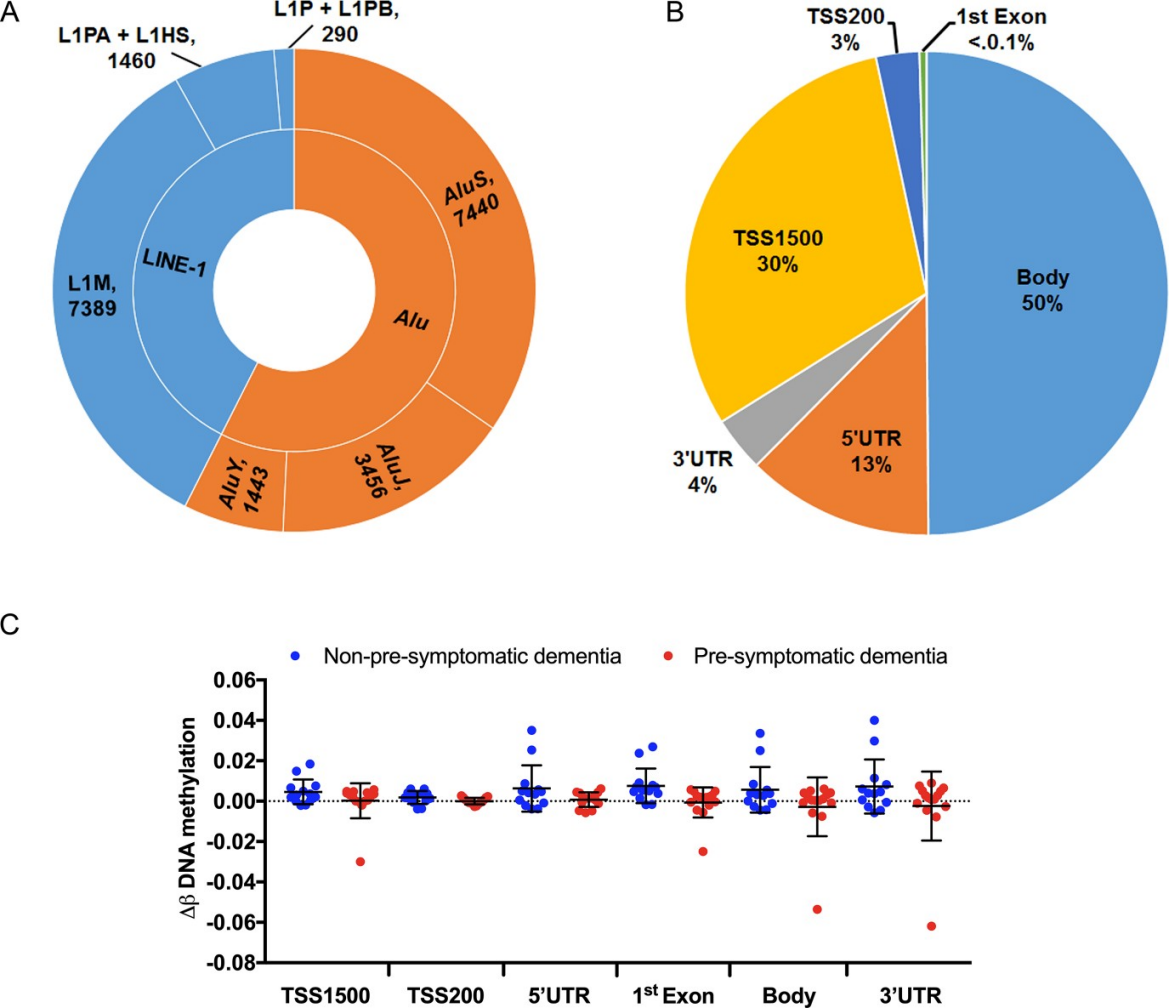
Distribution and methylation changes of LINE-1 and *Alu* probes. (A) Number of total probes by evolutionary age of LINE-1 and *Alu* elements. (B) Number of probes by genomic location. (C) Alterations in DNA methylation of retrotransposons at the 18-month follow-up in non-pre-symptomatic dementia and pre-symptomatic dementia by genomic location.

A total of 9,139 probes mapping to LINE-1 elements and 12,339 probes mapping to *Alu* sequences on the 450K array were analysed in relation to PSD. Of the LINE-1 probes, 7,389 map to L1M (old) elements, 1,460 to L1P and L1PB (intermediate) elements, and 290 to L1HS and L1PA (youngest) elements (Fig 2A). Of the *Alu* probes, 3,456 map to *Alu*J (oldest) elements, 7,440 map to *Alu*S (intermediate) elements, and 1,443 to *Alu*Y (youngest) elements. The majority of the interrogated repetitive element loci were located within gene bodies (50%), while 30% were in TSS1500 and 3% in TSS200 (Fig 2B); the rest of the elements were located in the 5’UTR (13%) and 3’UTR (4%). DNA methylation across all retrotransposons by genomic location did not significantly differ between PSD and non-PSD cases (Fig 2C).

### DNA methylation changes within LINE-1 and *Alu* elements in PSD

**Fig 3:**
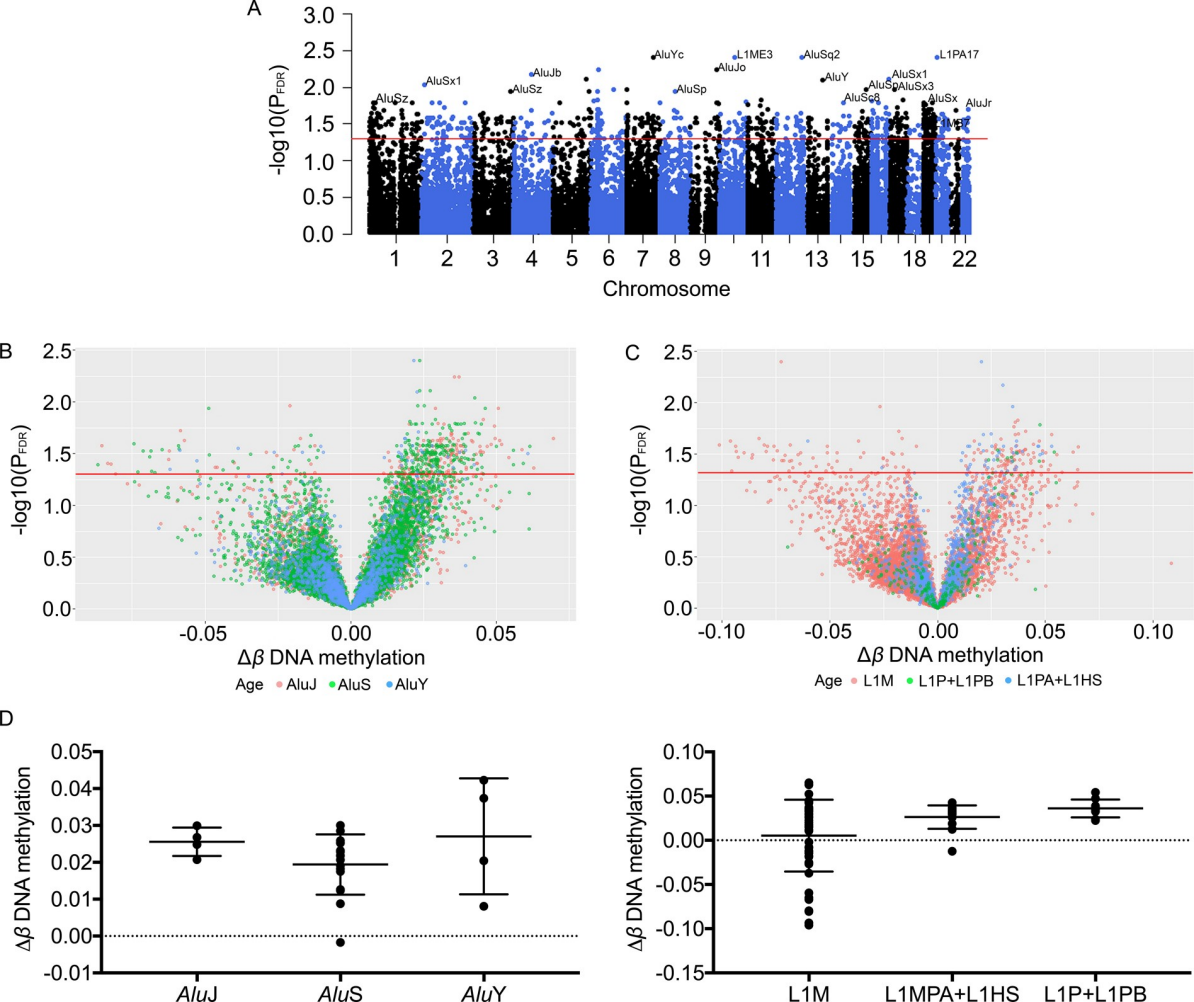
Differentially methylated positions in pre-symptomatic dementia (PSD) at 18-month follow-up. (A) Manhattan plot of retrotransposon loci displaying significant changes in DNA methylation between type 2 diabetes patients with and without PSD at follow-up. B-C) Volcano plots of changes in retrotransposon methylation (Δ*β*) in PSD by evolutionary age of *Alu* (B) and LINE-1 (C) elements. D) Changes in DNA methylation (Δ*β*) by evolutionary age of LINE-1 and *Alu* elements. The Red line represents P_**FDR**_ = 0.05.

Changes in DNA methylation of retrotransposons at the 18-month follow-up compared with baseline were examined for all participants. Among the 21,478 of retrotransposon loci, there were 714 significant differentially methylated positions (DMPs; P_FDR_ < 0.05) in PSD at follow-up (Fig 3A and S2 Table), corresponding to 2.4% of all LINE-1 and 3.8% of all *Alu* probes analysed, and enriched significantly within the *Alu*J and *Alu*S families (p *<* 0.0001). Of these, only three probes were also significantly different in non-PSD subjects at follow-up. The majority of the loci were hypermethylated (424 *Alu* loci and 155 LINE-1 loci; P_FDR_ < 0.05), while 62 *Alu* loci and 73 LINE-1 loci were significantly hypomethylated (P_FDR_ < 0.05) (Fig 3B and 3C). The DNA methylation changes (follow-up *vs* baseline) by retrotransposon subfamily evolutionary age are shown in Fig 3D. The oldest LINE-1 subfamilies (L1M) demonstrated the largest variation in magnitude of DNA methylation changes (Δ*β*) within LINE-1 elements [standard deviation (SD) = 0.041] and tended to be hypomethylated at the 18-month follow-up, while the youngest *Alu* subfamilies (*Alu*Y) had the greatest variation in methylation (SD = 0.016) of all *Alu* elements (Fig 3D). We did not observe changes in DNA methylation at non-retrotransposon probes mapping in close proximity to the DMPs, suggesting that these changes are highly loci specific.

### Gene functions and pathway analysis

**Fig 4:**
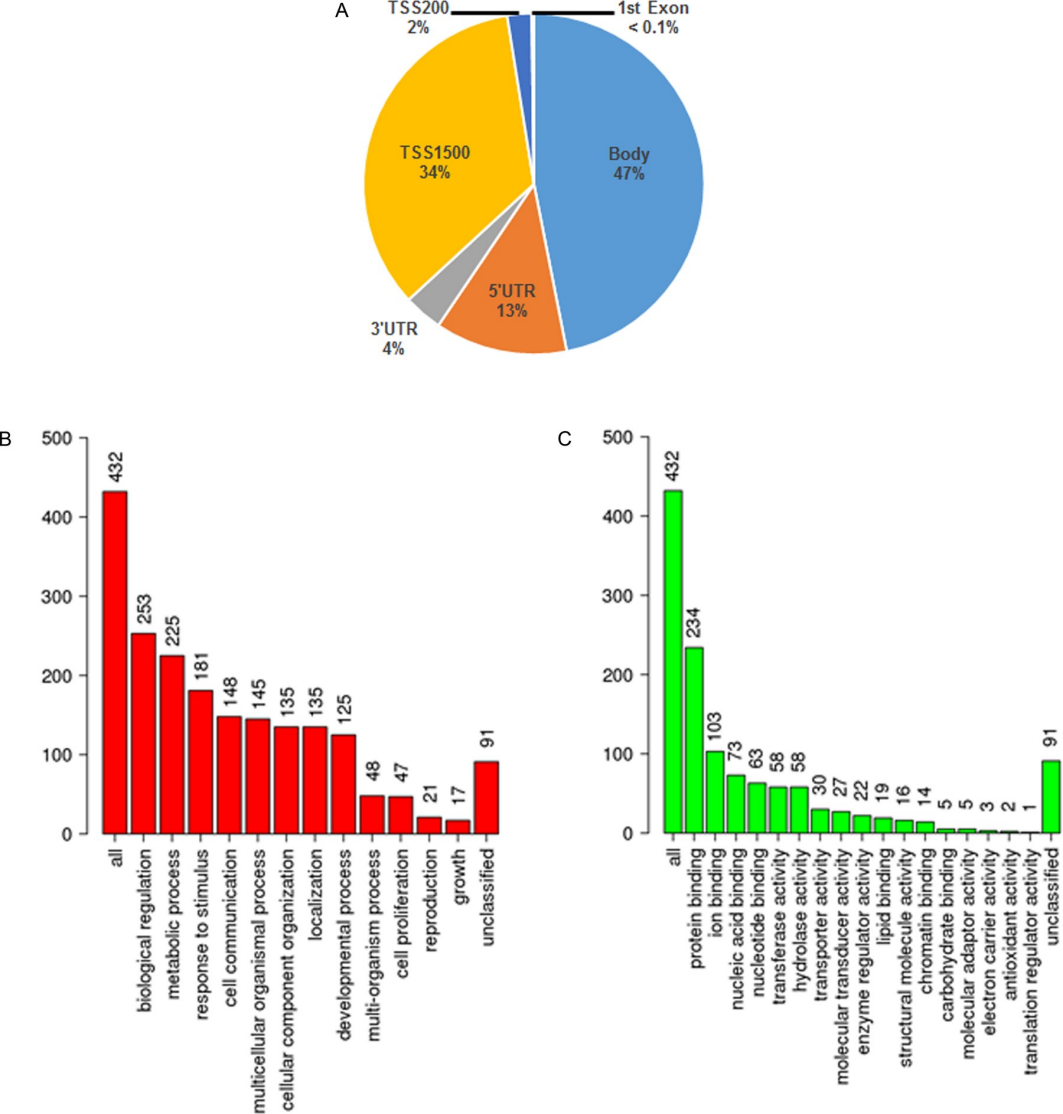
Pathway analysis for the 714 differentially methylated positions. (A) Number of probes by genomic location. B-C) Pathway analysis for the 432 genes associated with the 714 DMPs by biological process categories (B) and molecular function categories (C).

The DMPs in PSD mapped to gene bodies (47% of total loci), TSS1500 (34%), 5’UTR (13%), 3’UTR (4%), TSS200 (2%) and 1^st^ Exon (< 0.1%), and demonstrated enrichment at TSS1500 regions (p = 0.013) (Fig 4A). Pathway analysis incorporating the DMPs showed that aberrantly methylated retrotransposon loci were involved in multiple pathways relating to dementia (Fig 4B and 4C); the most significant pathway (by p value) was the muscarinic acetylcholine receptor 1 and 3 signalling pathway (p = 0.007), which is implicated in AD development and cognitive decline. Additionally, the Alzheimer disease-amyloid secretase (p = 0.012), metabotropic glutamate receptor group 3 and group 1 (p = 0.015 and p = 0.017, respectively), beta 3 adrenergic receptor signalling (p = 0.019), interferon-gamma signalling (p = 0.025), corticotropin releasing factor receptor signalling (p = 0.028), apoptosis signalling (p = 0.030), 5HT4 type receptor mediated signalling (p = 0.030) and opioid proenkephalin (p = 0.030) pathways were also significantly enriched using Over Representation Analysis in WebGestalt (S3 Table).

**Fig 5:**
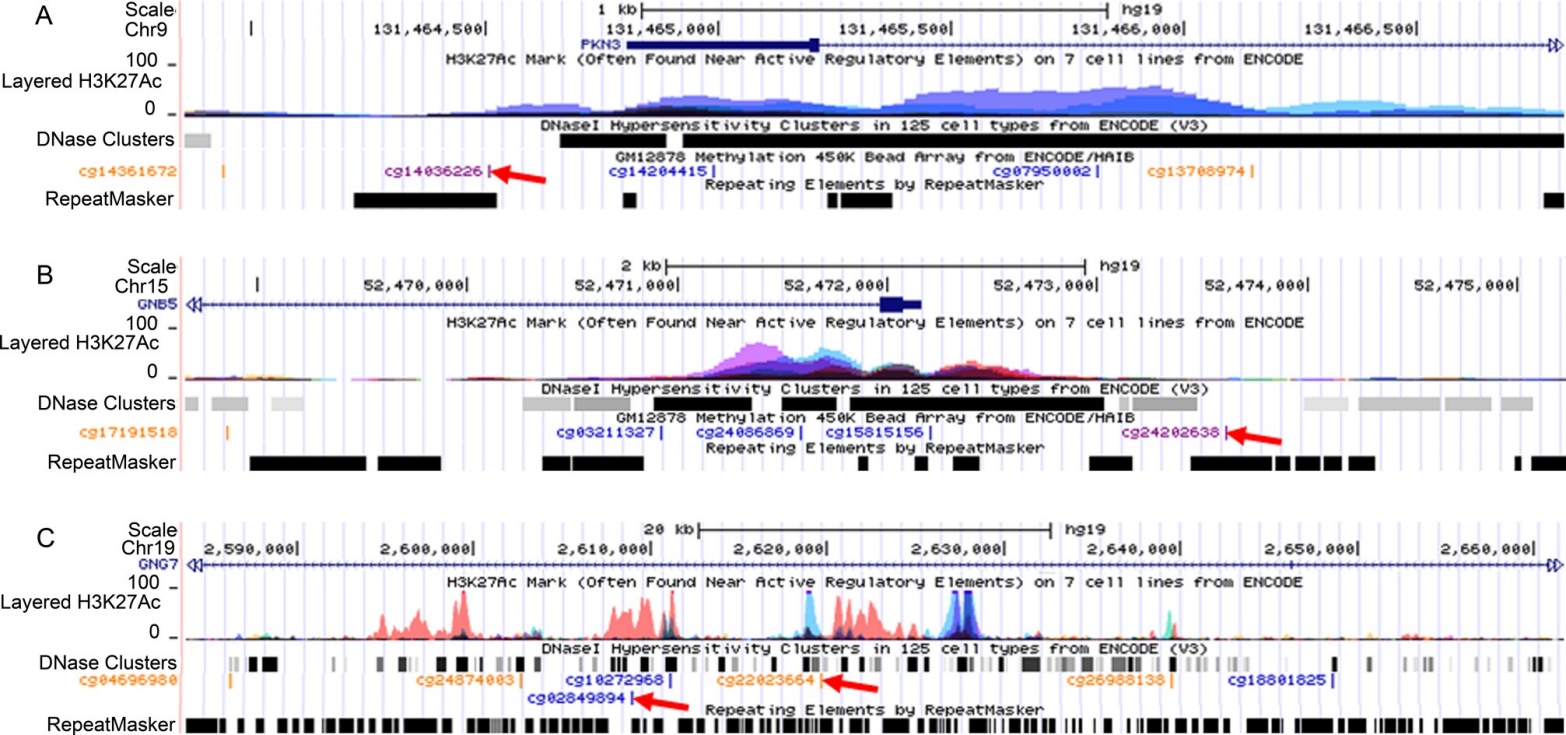
Genomic location of significant differentially methylated retrotransposons. (A) Protein Kinase N3 (*PNK3*). (B) G Protein Subunit β 5 (*GNB5*). C) G Protein Subunit γ 7 (*GNG7*). Red arrows indicate DMPs identified by the study.

Within pathways related to dementia development, *Alu*S elements mapping to TSS1500 regions within the Protein Kinase N3 (*PKN3*; cg14036226) and G Protein Subunit β 5 (*GNB5*; cg24202638) genes (Fig 5A and 5B) and L1M elements (cg22023664 and cg02849894) within the 5’UTR of G Protein Subunit γ 7 (*GNG7*) (Fig 5C) were differentially methylated.

### The effects of folic acid and vitamin B_12_ supplementation on DNA methylation at DMPs of PSD

**Fig 6:**
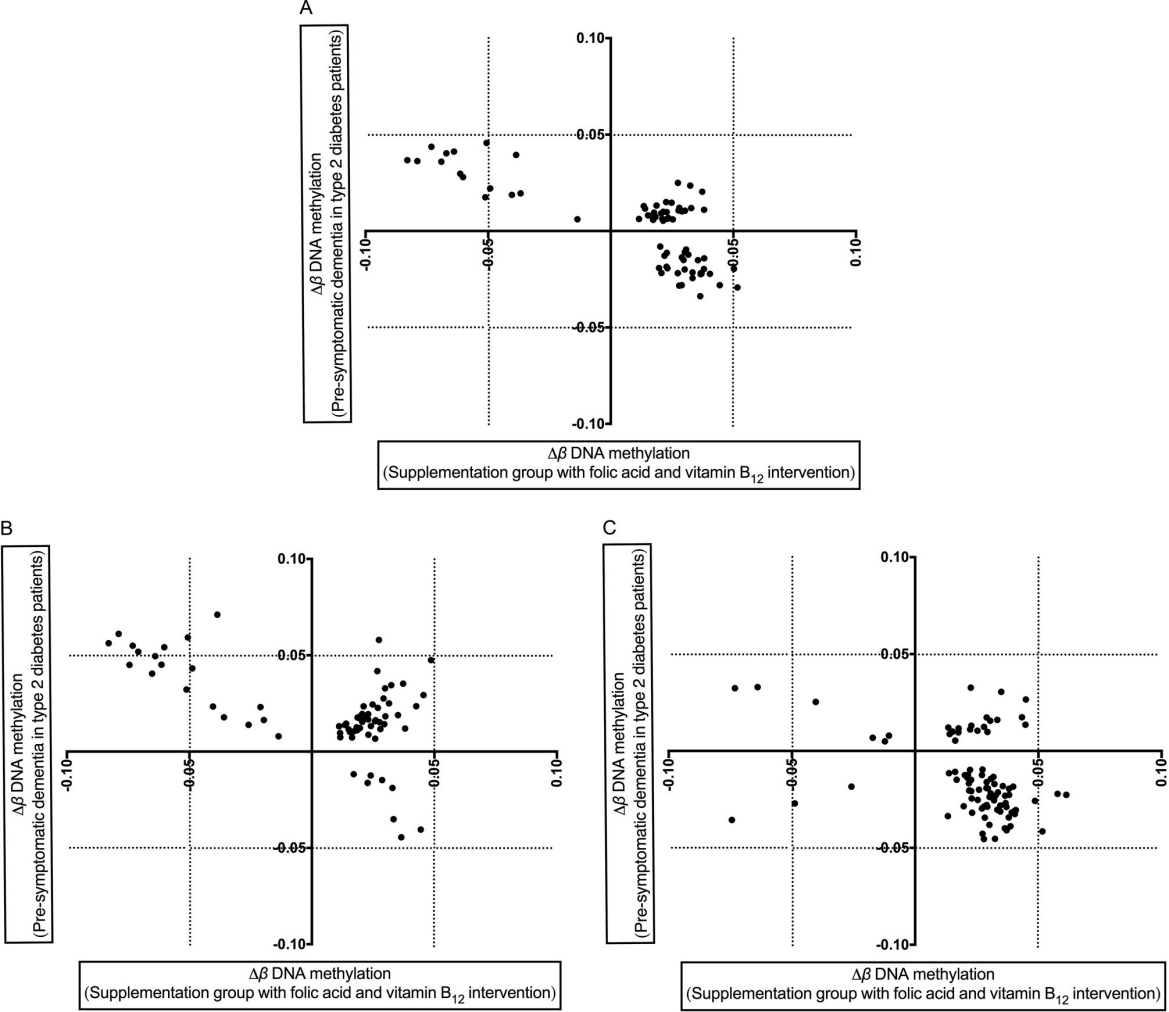
DNA methylation changes between B-vitamins intervention and pre-symptomatic dementia development. Changes in DNA methylation (Δ*β*) in the two studies are presented in all participants (A) and stratified by those healthy individuals in the intervention study with the *MTHFR* 677CC (B) and TT (C) genotypes. X-axis represents Δ*β* DNA methylation of pre-symptomatic dementia in T2D patients and Y-axis represents Δ*β* DNA methylation of supplementation group with folic acid and vitamin B_**12**_ intervention.

To assess the effects of supplementation with folic acid and vitamin B_12_ on DNA methylation of the 714 PSD-associated DMPs, an independent intervention study dataset was used (supplementation group: *n* = 44; placebo group: *n* = 43). DNA methylation at 85 of the 714 loci were significantly altered (P_FDR_ < 0.05) following the two-year intervention. An additional 12 loci which overlapped with the placebo group were excluded from further analysis. Methylation at 15 DMPs, nine of which mapped to L1M elements that were hypomethylated in the PSD group, significantly increased (P_FDR_ < 0.05) after folic acid and vitamin B_12_ supplementation (Fig 6A, top-left quarter and Table 1). Four of these 15 loci were also related to AD: cg14051544 of HAL1-2a-MD in the 5’UTR of Solute Carrier Family 7 Member 14 (*SLC7A14*); cg22023664 of L1M in the 5’UTR of *GNG7*; cg23342367 of L1M in the gene body of Tumor Protein D52 (*TPD52*); and cg23304647 of L1M in the gene body of G Protein Subunit Alpha 12 (*GNA12*). An additional 29 probes within *Alu*J (35%), *Alu*S (24%), *Alu*Y (7%), of L1M (24%), L1PA (7%) and L1PB (1%) elements were positively correlated with the development of PSD and displayed significantly decreased DNA methylation (paired t-test, P_FDR_ < 0.05) following dietary intervention (Fig 6A, bottom-right quarter and S4 Table). However, another 29 loci were hypermethylated both in the vitamin-supplemented and PSD groups (Fig 6A, top-right quarter and S5 Table). When examined by *MTHFR* genotypes (677CC and 677TT), DNA methylation levels of 71 of the 714 PSD-associated DMPs were significantly altered in participants with the *MTHFR* CC genotype (8 hypomethylated, 19 hypermethylated) (Fig 6B), whereas 89 loci were significantly different in participants carrying the *MTHFR* TT genotype (61 hypomethylated, 6 hypermethylated) after intervention (Fig 6C).

**Table 1:** Hypermethylated CpG sites after folic acid and vitamin B_12_ supplementation.

Probe ID	Retrotransposon	Gene	Genomic location	CpG density	Gene function	Relation to Alzheimer’s disease
**cg03316030**	*AluJ*	*LTB*	TSS1500	S_Shelf	Signalling receptor binding and tumour necrosis factor receptor binding	NA
**cg05310486**	*AluS*	NA	NA	N_Shelf	NA	NA
**cg20227511**	*AluS*	*FLT3*	Body	N_Shelf	Protein homodimerisation activity and protein kinase activity	NA
**cg27324781**	*AluY*	NA	NA	S_Shelf	NA	NA
**cg14051544**	HAL1-2a_MD	*SLC7A14*	5'UTR	N_Shore	Amino acid transmembrane transporter activity and L-amino acid transmembrane transporter activity	Expressed in OXYS rat at the early stage of Alzheimer’s disease
**cg06305891**	L1M	NA	NA	NA	NA	NA
**cg15980656**	L1M	*SDC2*	Body	NA	PDZ domain binding and cytoskeletal protein binding	NA
**cg22023664**	L1M	*GNG7*	5'UTR	N_Shelf	Obsolete signal transducer activity	Marker of pre-symptomatic dementia
**cg23342367**	L1M	*TPD52*	Body	NA	Calcium ion binding and protein heterodimerisation activity	Neuron related genes
**cg23911433**	L1M	NA	NA	NA	NA	NA
**cg23304647**	L1M	*GNA12*	Body	S_Shelf	GTP binding and obsolete signal transducer activity	Upregulated in late-onset Alzheimer’s disease
**cg13755866**	L1M	NA	NA	NA	NA	NA
**cg27609217**	L1M	NA	NA	N_Shore	NA	NA
**cg12785183**	L1M	NA	NA	NA	NA	NA
**cg14567424**	L1PA	*GSTP1*	TSS1500	N_Shore	Glutathione transferase activity and kinase regulator activity	NA

NA: not available

A threshold of Δ*β* value = ±0.05 was used to identify loci displaying substantial changes in methylation levels following dietary supplementation. For all participants, methylation at 10 repetitive element loci increased significantly while one decreased significantly following supplementation (S6 Table), which corresponded to DMPs that were hypomethylated (10 loci) and hypermethylated (1 locus) in PSD. With both the CC and TT genotypes of *MTHFR*, methylation of cg05310486 (*Alu*S) and cg23911433 (L1M) increased by Δ*β* > 0.05. However, in participants with the CC genotype, methylation of 10 other loci (cg27324781, cg23304647, cg14567424, cg15980656, cg26381210, cg12785183, cg10959668, cg24534583, cg06305891, and cg23342367), increased by Δ*β* > 0.05 after vitamin supplementation and had lower methylation within the PSD group, while methylation of cg01168028 was increased by supplementation and within PSD patients (Fig 6B). In those with the TT genotype only, methylation of three CpG loci (cg23489236, cg10675515, and cg27133230) decreased by Δ*β* > 0.05 following dietary supplementation and methylation at these loci correlated inversely with DNA methylation changes in PSD. In addition, methylation of cg07291836 decreased both in the PSD group and after intervention (Fig 6C).

### Association between differentially methylated retrotransposon loci and serum/plasma concentrations of B-vitamins and homocysteine

**Fig 7:**
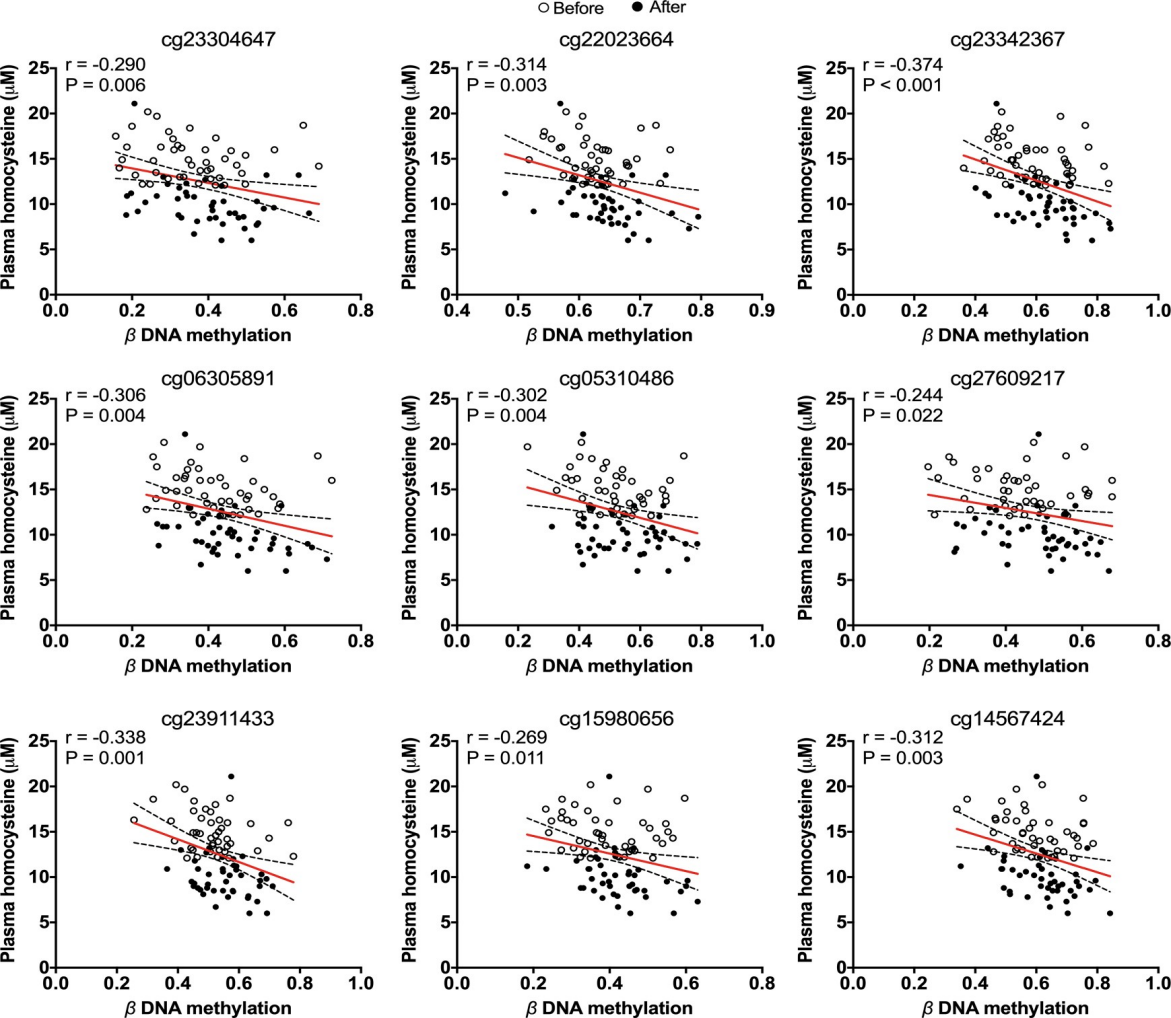
Correlation of DNA methylation and plasma homocysteine levels in healthy participants.

We investigated the relationship between DNA methylation of the 15 significantly hypermethylated DMPs and concentrations of serum folate, vitamin B_12_ and plasma homocysteine in participants in the intervention study. We observed inverse correlations between DNA methylation and homocysteine concentration for 9 of the 15 loci displaying hypermethylation after supplementation with B-vitamins; cg23304647 (r = -0.290, p = 0.006), cg22023664 (r = -0.314, p = 0.003), cg23342367 (r = -0.374, p < 0.001), cg06305891 (r = -0.306, p = 0.004), cg05310486 (r = -0.302, p = 0.004), cg27609217 (r = -0.244, p = 0.022), cg23911433 (r = -0.338, p = 0.001), cg15980656 (r = -0.269, p = 0.011), cg14567424 (r = -0.312, p = 0.003) (Fig 7). Moreover, at six of these nine loci, there were positive correlations between methylation and serum vitamin B_12_ concentration; cg22023664 (r = 0.212, p = 0.048), cg23342367 (r = 0.222, p = 0.037), cg06305891 (r = 0.223, p = 0.037), cg05310486 (r = 0.219, p = 0.040), cg27609217 (r = 0.244, p = 0.022), cg14567424 (r = 0.211, p = 0.048) (S1 Fig). There were no significant correlations between methylation and serum folate concentration.

## Discussion

In this study, we identified 714 loci mapping to retrotransposons (LINE-1 and *Alu*) that were differentially methylated in T2D patients with PSD compared to those with normal cognitive function, with such changes enriched within older families (L1M, *Alu*J, and *Alu*S). LINE-1 elements are enriched within intergenic regions of the genome while *Alu* elements are preferentially integrated upstream of genes and within introns [61], but for both there are extensive reports on their impact upon gene expression via cis- and trans-regulation. In concordance with their genomic distribution, the probes on the Illumina Infinium 450K mapping to retrotransposons predominantly correspond to TSS1500, gene bodies and intergenic regions, with relative depletion close to transcriptional start sites and within exons [20]. As changes at those probes mapping to TSS1500 are more likely to have a functional impact upon gene expression, such as through promoter hypomethylation, we observed enrichment of DMPs at TSS1500. This finding supports the hypothesis that changes in retrotransposon DNA methylation may have influence through mediation of neighbour gene expression. Further, we observed that a number of PSD-associated DMPs showed inverse changes in methylation with folic acid and vitamin B_12_ supplementation. To our knowledge, this is the first study to analyse methylation of retrotransposons by their evolutionary age in people with T2D-associated PSD and in response to dietary intervention.

Changes in methylation of LINE-1 or *Alu* elements during development and ageing may cause DNA damage and double-strand DNA breaks that contribute to the process of neurodegeneration [62, 63]. Moreover, our locus-specific analysis revealed changes in elements mapping to genes associated with dementia including *GNB5*, *GNG7*, and *PKN3*. Specifically, we have identified hypermethylation of an *Alu*Sx element at the 5’ region of the *GNB5* promoter, and an *Alu*Sg element within the *PKN3* promoter. We observed enrichment of DMPs within *Alu*J and *Alu*S sequences. While the reasons for this are not currently clear, it may suggest that such elements are implicated in PSD not through retrotransposition, but rather through modifying the expression of proximal genes. Younger repetitive element subfamilies such as L1HS and *Alu*Y are known to be more active in retrotransposition [12, 64], and therefore the differential methylation of *Alu*J and *Alu*S elements are unlikely to result in somatic retrotransposition events. Loss of *GNG7* is associated with dysregulation of the adenylyl cyclase signalling pathway that is associated with a number of neurodegenerative disorders [65]. *GNB5* is similarly implicated in regulation of adenylyl cyclase signalling, and genetic variants within the gene are associated with impaired cognition [66], while *Gnb5* knockout mice display severe impairment of development and motor co-ordination [67]. PKN3 activity increases in response to insulin signalling [68] and may have a neuroprotective effect in response to hypoxia [69]. Furthermore, PKN family members have previously been implicated in the biology of AD [70]. Our study has therefore identified epigenetic changes within retrotransposons mapping to genes with critical functions in neuronal biology and the development of neurodegenerative disorders. However, further functional studies will be required to examine the subsequent impact upon the expression of these genes and fully elucidate the role of retrotransposons in dementia biology.

Genome-wide hypomethylation of LINE-1 and *Alu* elements causes loss of genomic integrity [71, 72]. Folic acid and vitamin B_12_ are key players in one-carbon metabolism which generates SAM [73, 74], the universal methyl group donor used for DNA methylation. We observed that these nutrients modified DNA methylation of retrotransposons, including PSD-associated DMPs. Although these nutrients increased methylation of some CpG sites within retrotransposons that are also hypermethylated in PSD (Fig 6), this effect was small for most of the analysed loci (*e*.*g*. the change in methylation of cg02276269 and cg19764326 was below 0.05). Interestingly, nine of the 15 loci displaying hypermethylation after supplementation with B-vitamins were negatively correlated with plasma homocysteine concentrations, while DNA methylation at six loci were positively correlated with serum vitamin B_12_ levels. Repetitive elements display differential sensitivity to environmental exposures [23], and it may be that epigenetic changes within repetitive elements in response to internal stimuli or environmental factors can serve to modify the expression of genes implicated in PSD development. DNA methylation of old LINE-1 subfamily (L1M) seems to be vulnerable to dietary exposure than younger subfamilies.

Additionally, we observed differences in retrotransposon DNA methylation by *MTHFR* genotype following dietary supplementation with folic acid and vitamin B_12_. Specifically, higher methylation levels were observed at some loci in in participants with the *MTHFR* CC genotype (the most common variant of *MTHFR*), whereas lower levels were observed at the same loci in participants with the *MTHFR* TT genotype. In those carrying the *MTHFR* TT genotype, MTHFR enzyme activity is lower leading to decreased SAM production and, potentially, resulting in hypomethylation events. Therefore, and in concurrence with our previous study [35], individuals with the *MTHFR* CC genotype may show greater benefit from supplementation with folic acid and vitamin B_12_ than those with the *MTHFR* TT genotype.

Our study has limitations. The number of participants in each of the two studies was relatively small (38 participants for analysis of changes in PSD and 44 participants receiving dietary supplementation). Despite the measures in the study design and our analysis to increase statistical power and limit the risk of yielding false positives, we cannot exclude the possibility that some of the DMPs identified in this study may be artefacts. Our results will need to be confirmed in independent cohorts, and the role of retrotransposon methylation in the initiation of dementia will need to be examined further through *in vitro* studies. Secondly, the data on effects of folic acid and vitamin B_12_ supplementation were obtained from healthy older people and not in T2D patients. Due to the absence of cognition data, we cannot exclude the possibility that some of the participants within the dietary supplementation study may have PSD; therefore, care must be taken in extrapolating the results to the development of PSD in those with T2D. However, both studies were undertaken in older people and the intervention and follow-up periods were of similar duration (*i*.*e*. one-and-a-half and two years respectively), the resulting data may be compatible.

To the best of our knowledge, there are no publicly-available RNA-seq datasets available to examine the link between altered DNA methylation and gene expression to understand the underlying mechanisms of PSD in T2D. Future studies may require such analysis to reveal the functionality of these DMRs in the aetiology of PSD in T2D patients.

## Conclusions

Our study is the first to report that locus-specific changes in DNA methylation within retrotransposons is associated with PSD incidence in T2D patients, and that the methylation of these sites might be modifiable by dietary supplementation with folic acid and vitamin B_12_. Further, we observed that the effects of these nutrients on DNA methylation differ by *MTHFR* genotype. If confirmed in future larger studies, these findings may contribute to the identification of epigenetic biomarkers of dementia risk in those with T2D and will strengthen the evidence for dietary intervention to reduce the risk of dementia among high-risk T2D patients.

## Supporting information

S1 Table Clinical dementia rating (CDR) in T2D with and without PSD. (XLSX)

S2 Table 714 significant differentially methylated positions. (XLSX)

S3 Table Pathway analysis. (XLSX)

S4 Table List of loci in Fig 6A, bottom-right quarter. (XLSX)

S5 Table List of loci in Fig 6A, top-right quarter. (XLSX)

S6 Table Overlapped loci between PSD patients and dietary intervention study. (XLSX)

S1 Fig (JPG)

## List of abbreviations

T2D: Type 2 diabetes mellitus
LINE-1: Long Interspersed Nuclear Element 1
AD: Alzheimer’s disease
PSD: Pre-symptomatic dementia
SAM: *S*-adenosylmethionine
MTHFR: Methylenetetrahydrofolate reductase
IDCD: Israel Diabetes and Cognitive Decline
CDR: Clinical dementia rating
SD: Standard deviation
DMP: Differentially methylated position
GNB5: G Protein Subunit β 5
GNG7: G Protein Subunit γ 7
PKN3: Protein Kinase N3
SLC7A14: Solute Carrier Family 7 Member 14
TPD52: Tumor Protein D52
GNA12: G Protein Subunit Alpha 12
