# Supplementary figures and images for: DNA methylation patterns of LINE-1 and Alu for pre-symptomatic dementia in type 2 diabetes
Source: PLoS One. 2020 Jun 11;15(6):e0234578. doi: 10.1371/journal.pone.0234578 (PMC7289438; doi:10.1371/journal.pone.0234578)

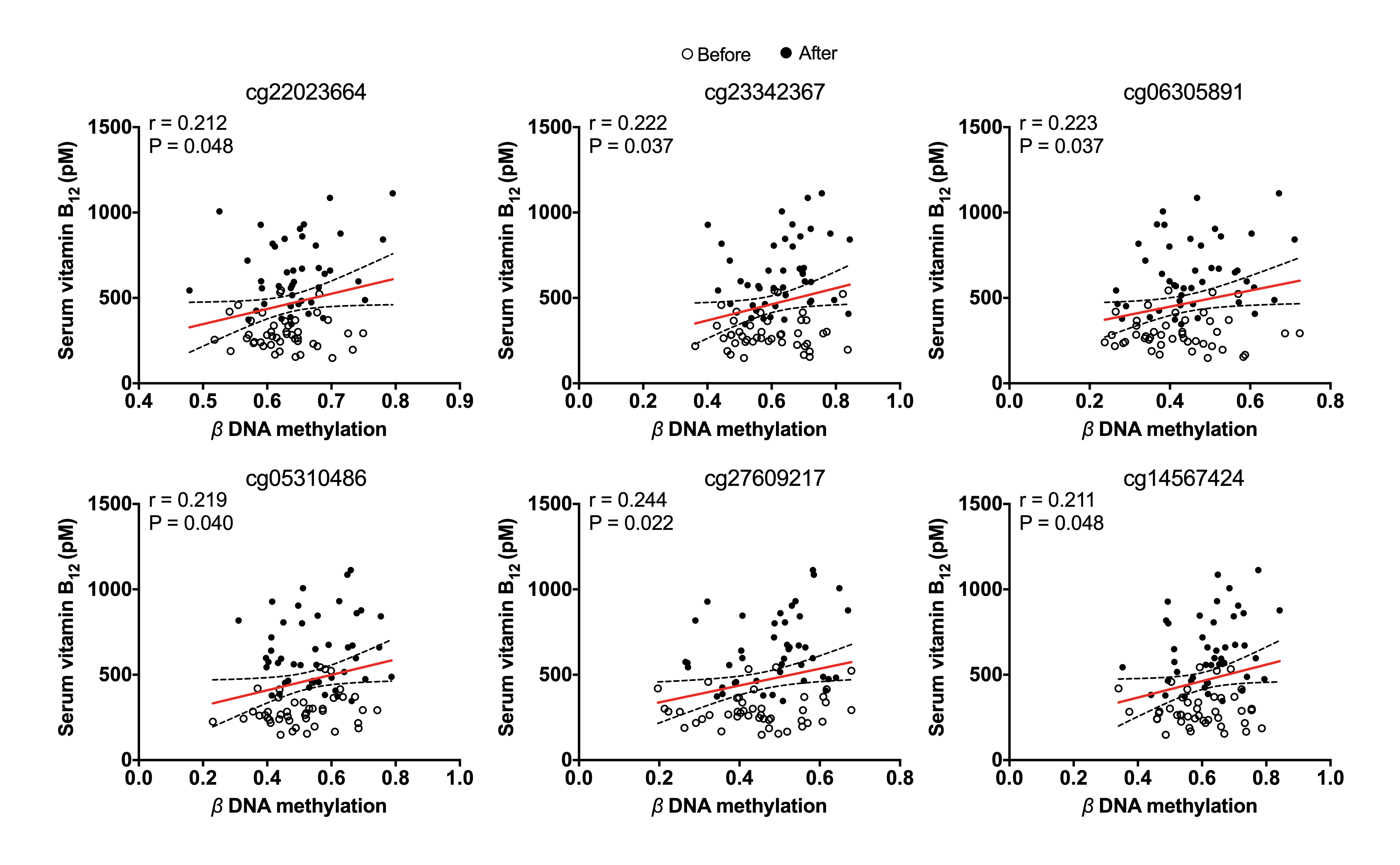

Supplement: S1 Fig — (JPG) [file pone.0234578.s007.jpg]
